# Pneumocystis Pneumonia and Disseminated Cryptococcosis Dual Infection: A Case Report

**DOI:** 10.7759/cureus.77538

**Published:** 2025-01-16

**Authors:** João Filipe Félix Vieira Afonso, Mafalda Maria Santos, Joana Vieira, Rafael Oliveira, Ana Filipa Rodrigues

**Affiliations:** 1 Internal Medicine, Unidade Local de Saúde do Oeste, Caldas da Rainha, PRT

**Keywords:** acute hemorrhagic stroke, cryptococcosis, disseminated cryptococcosis, hiv/aids-related opportunistic infections, hiv diseases, pneumocystis jiroveci pneumonia

## Abstract

Human immunodeficiency virus (HIV) is a virus that targets and destroys a cluster of differentiation 4 (CD4) cells. When left untreated, HIV can lead to acquired immunodeficiency syndrome (AIDS), which the presence of AIDS-defining diseases can characterize. Here, the authors report the diagnostic and therapeutic approach to a case of a 56-year-old male living with HIV who presented to the emergency department with dyspnea and fever. Chest CT revealed ground-glass opacities, consistent with *Pneumocystis *pneumonia (PCP)*. *Bronchoalveolar lavage culture confirmed PCP, and *Cryptococcus neoformans* was also identified. Lumbar puncture and MRI endorsed the diagnosis of disseminated cryptococcosis. The patient underwent targeted treatment for both opportunistic infections. However, after completing the therapeutic scheme, his neurologic condition deteriorated, and cerebral CT revealed hemorrhagic lesions, necessitating transfer to a neurosurgery unit.

## Introduction

The United States Centers for Disease Control and Prevention (CDC) defines acquired immunodeficiency syndrome (AIDS) as a cluster of differentiation 4 (CD4) cell count below 200/microL or the presence of any AIDS-defining condition regardless of CD4 cell count [1]. AIDS-defining conditions occur more frequently and with greater severity due to the immunosuppression caused by human immunodeficiency virus (HIV) infection [2]. The CDC includes several diseases (infections, neoplasms, and other conditions) as part of AIDS-defining conditions [2]. Opportunistic infections usually occur when CD4 cell count falls below 200 cells/microL [3]. In patients without antiretroviral therapy (ART), it takes approximately 12-18 months to develop an AIDS-defining condition once the CD4 cell count is less than 200 cells/microL [4]. Before ART, AIDS-defining conditions were the main cause of morbidity and mortality in people living with HIV [5]. However, for patients with late-stage HIV diagnoses, non-adherence to ART, and advanced HIV infection (CD4 <50 cells/microL), the median survival time is 12-18 months [6].

*Pneumocystis jirovecii*, a yeast-like fungus, causes *Pneumocystis *pneumonia (PCP). Despite a reduction in incidence due to ART, *Pneumocystis jirovecii* is still one of the main causes of opportunistic infection in HIV-infected patients, with an incidence of <1 case per 100 person-years [7]. Ninety percent of cases occur in people with CD4 <200 cells/microL, with the main risk factor being advanced immunosuppression in patients not taking ART. Other risk factors include CD4 cell count <200 cells/microL, CD4 cell percentage <14%, previous episodes of PCP, mucocutaneous candidiasis, recurrent bacterial pneumonia, unintentional weight loss, and high HIV RNA viral load [8]. PCP has a gradual onset, typically presenting with fever (80% to 100%), non-productive cough (95%), and dyspnea (95%) that worsens within days to weeks [9]. Hypoxemia, ranging from mild (alveolar-arterial O2 difference <35 mmHg) to severe (alveolar-arterial O2 difference >45 mmHg), lactate dehydrogenase (LDH) >500 mg/dL, and elevated 1,3-beta-D-glucan may be observed despite being non-specific [10]. Diffuse, interstitial, or alveolar infiltrates are the most common chest radiographic findings, although nodules, blebs and cysts, upper lobe involvement, intrathoracic adenopathy, and spontaneous pneumothorax can also be seen [11]. High-resolution CT often shows bilateral ground-glass opacities [12]. Definitive diagnosis requires histopathologic and microbiologic demonstration of *Pneumocystis jirovecii* in sputum, bronchoalveolar lavage (BAL) fluid, or biopsy via polymerase chain reaction (PCR) or staining [10]. Empiric treatment must be initiated promptly, regardless of definitive diagnosis, as PCP should be strongly suspected in an untreated HIV patient with CD4 cell count <200 cells/microL who presents the classic symptoms and interstitial or alveolar infiltrates on chest radiograph or high-resolution CT [13]. CDC guidelines recommend trimethoprim-sulfamethoxazole (trimethoprim 15-20 mg/kg/day and sulfamethoxazole 75-100 mg/kg/day, in divided doses every six or eight hours) for 21 days. Patients with moderate-to-severe disease (room air PaO2 <70 mmHg or alveolar-arterial gradient ≥35 mmHg) should receive adjunctive corticosteroids as soon as possible. ART should be initiated within two weeks of PCP diagnosis [8]. PCP survival has improved with ART and directed therapy from 40% to 63% in recent years [14].

*Cryptococcus neoformans* infection is another severe opportunistic infection that occurs in AIDS patients. Ninety percent of cryptococcosis occurs in patients with CD4 counts <100 cells/microL [8]. Cryptococcal infection typically begins in the lungs and disseminates to the central nervous system (CNS) in most cases [15]. Pulmonary symptoms include dyspnea, cough, chest pain, and hemoptysis, ranging from asymptomatic to acute respiratory failure, with its severity being inversely proportional to CD4 cell count [15]. The most common radiographic sign is a solitary noncalcified nodule, although interstitial infiltrates mimicking PCP may also be seen [15]. Diagnosis requires sputum or BAL cultures, followed by blood cultures and CSF cryptococcal antigens and cultures if positive [16]. CNS is the most frequently affected system by *Cryptococcus neoformans*, presenting as meningitis or meningoencephalitis, characterized by fever, malaise, neck stiffness, photophobia, and headache, which occur in a two-week period. In more severe cases, it can progress to encephalopathy and coma due to hydrocephalus [8]. One of the main neurological complications of cryptococcal meningitis is lacunar strokes, especially in the basal ganglia [17]. Disseminated cryptococcosis may also involve the skin (papules, plaques, purpura, ulcers, cellulitis, and abscesses), liver, peritoneum, urogenital tract, and eyes [18]. Neuroimaging with CT or MRI prior to lumbar puncture can identify mass lesions, increased intracranial pressure, and hydrocephalus [19]. CFS analysis (characterized by low white blood cell counts with a mononuclear predominance, mildly elevated protein levels, and low-to-normal glucose concentrations), culture, and microscopy with India ink preparation (round encapsulated yeast forms can be seen in 60% of cases), cryptococcal antigen (a positive result in the CSF strongly suggests cryptococcosis, being sufficient to initiate treatment), and PCR can be used for cryptococcosis diagnosis [8]. CDC’s treatment guidelines for CNS and/or disseminated cryptococcosis consist of three phases: induction (liposomal amphotericin B 3-4 mg/kg intravenously once a day plus flucytosine 25 mg/kg orally four times a day for two weeks; lumbar puncture should be performed on days 7 and 14 of treatment to assess clinical response and culture sterility), consolidation (fluconazole 800 mg orally daily for at least eight weeks), and maintenance therapy (fluconazole 200 mg orally once a day for at least one year from initiation of antifungal therapy). ART initiation is deferred for four to six weeks after antifungal therapy since it has been associated with decreased survival and increased risk of immune reconstitution inflammatory syndrome (IRIS) [8]. The prognosis for patients with CNS cryptococcosis has improved in recent years due to antifungal treatment and ART; however, acute mortality has gone from 6% to 16% [20].

## Case presentation

A 56-year-old man presented to the emergency department with gradual onset dyspnea accompanied by cough and fever. His medical history included chronic obstructive pulmonary disease due to smoking (20 packs/year) and HIV infection without any follow-up or treatment, diagnosed three years prior during hospitalization for pulmonary actinomycosis. He did not have any recollection of when the transmission occurred.

**Figure 1:**
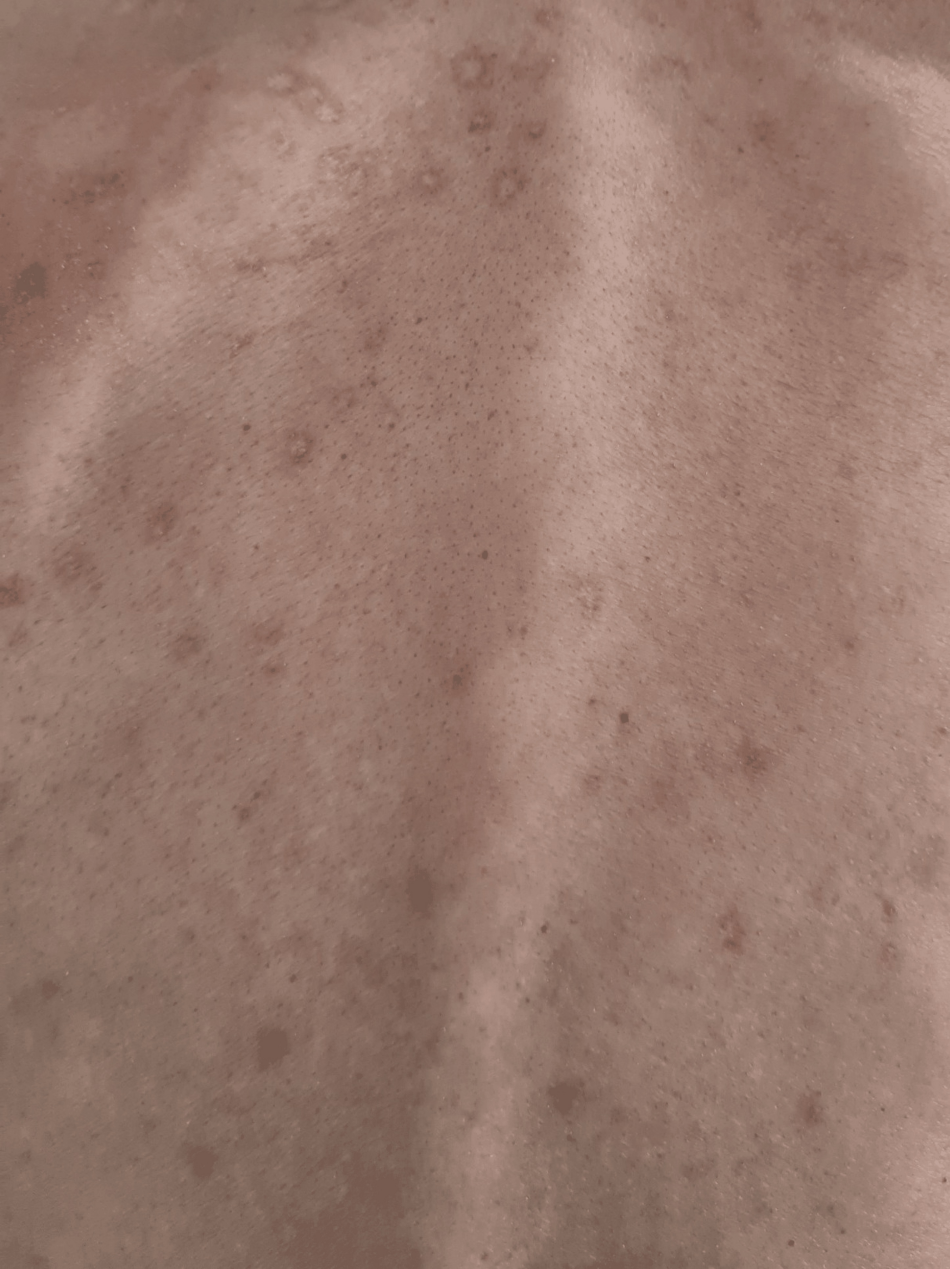
Papular lesions in the healing phase on the patient's back

On admission, his vital signs included blood pressure of 110/56 mmHg, heart rate of 110 beats per minute, and SpO2 of 82% (room air). He was conscious, with a Glasgow coma scale ranking of 15 (ocular response four points, verbal response five points, motor response six points), without changes in pupil size or shape. Other notable findings during the physical exam included white lesions on the tongue, palate, and oropharynx, compatible with oral candidiasis, and papular lesions in the healing phase in his back (Figure 1). No lymphadenopathy was noted. He was sarcopenic, weighing 50 kg. Lung auscultation revealed scattered crackles. The neurological exam was normal, and no meningeal signs were present.

Arterial blood gas (oxygen at two liters/minute) showed pH 7.45, PₐO₂ 73 mmHg, PₐCO₂ 39 mmHg, HCO₃- 27 mEq/L, and lactate 1.1 mmol/L, with an alveolar-arterial O2 gradient of 77.9 mmHg. Admission’s laboratory notable findings included leukocytes 4.5 x 10^3^/microL (lymphocytes 0.6 x 10^3^/microL), LDH 330 U/L, and C-reactive protein (CRP) 7.9 mg/dL (Table 1). The patient had 1 CD4+ cell/uL (normal 410-1590 cells/uL), a T-helper/suppressor ratio of 0, and an HIV viral load superior to 92,000 copies. Additional serologic studies for syphilis, *Toxoplasma gondii, Leishmania, *and Hepatitis B and C virus were negative. *Streptococcus pneumoniae* and *Legionella* urine antigen tests were also negative.

**Table 1:** Laboratory results of the patient during admission CRP: C-reactive protein, ESR: erythrocyte sedimentation rate, AST: aspartate aminotransferase, ALT: alanine transaminases, GGT: gamma-glutamyl transferase, LDH: lactate dehydrogenase

Parameter	Patient value	Normal range
White blood count	12.6 x 10^3^ u/L	4.0-10.0 X10^3^ u/L
Hemoglobin	13.1 g/L	13.6-18.0 g/L
CRP	7.9 mg/dL	<0.5 mg/dL
ESR	108 mm/hour	12-14 mm/hour
Total bilirubin	0.3 mg/dL	0.2-1.2 mg/dL
AST	17 U/L	5-34 U/L
ALT	20 U/L	0-55 U/L
GGT	30 U/L	12-64 U/L
Alkaline phosphatase	53 U/L	40-150 U/L
LDH	330 U/L	125-220 U/L
D-dimers	3823 ng/mL	<500 ng/mL
Lipase	4363 U/L	8-78 U/L
Calcium	9.1 mg/dL	8.4-10.2 mg/dL
Glucose	102 mg/dL	70-105 mg/dL
Creatinine	1.25 mg/dL	0.7-1.3 mg/dL
Urea	85 mg/dL	19-44 mg/dL
Procalcitonin	0.16 ng/dL	<0.5 ng/dL

**Figure 2:**
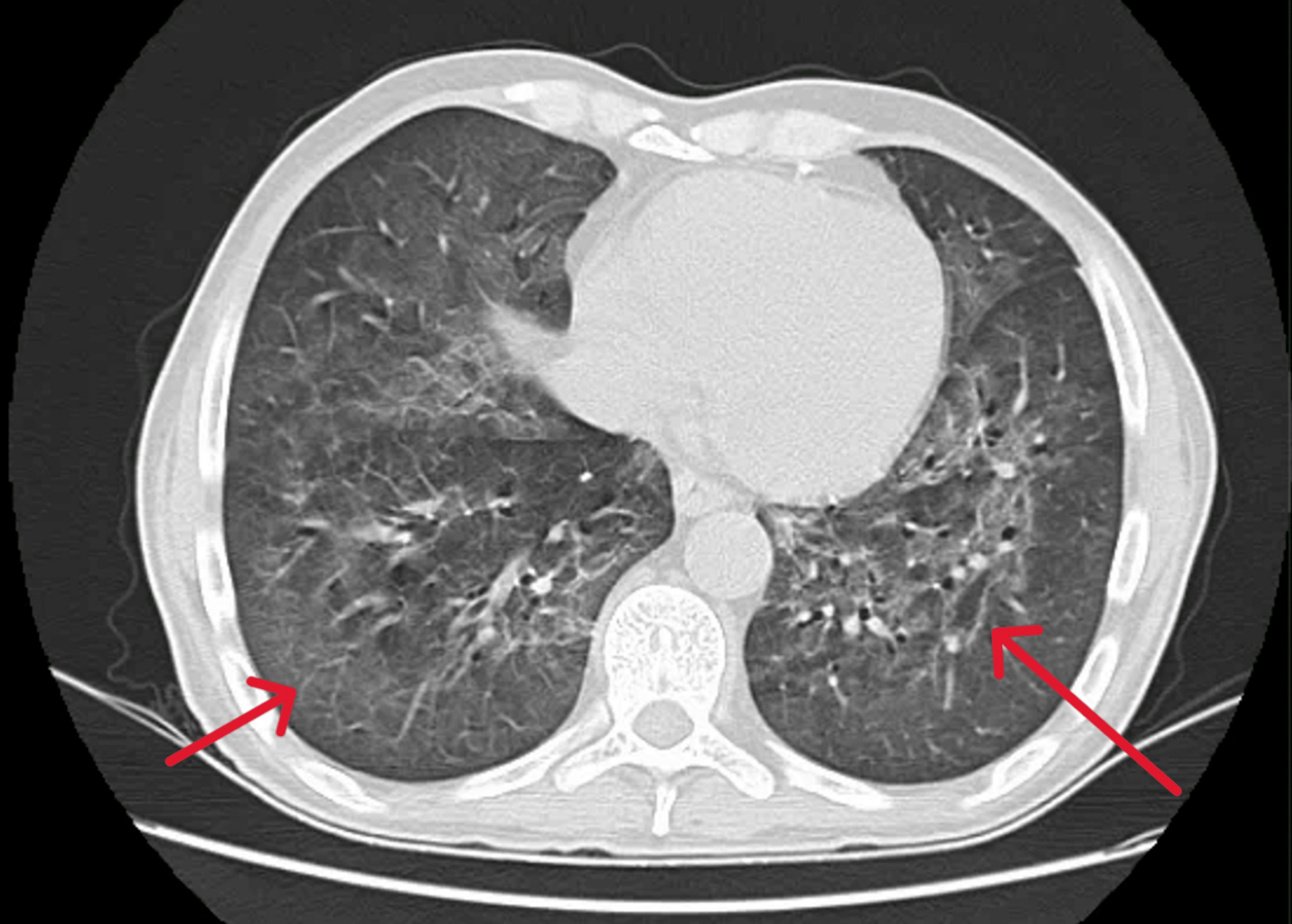
High-resolution CT scan of the lungs showing the bilateral ground-glass opacities (red arrows) CT: computed tomography

**Figure 3:**
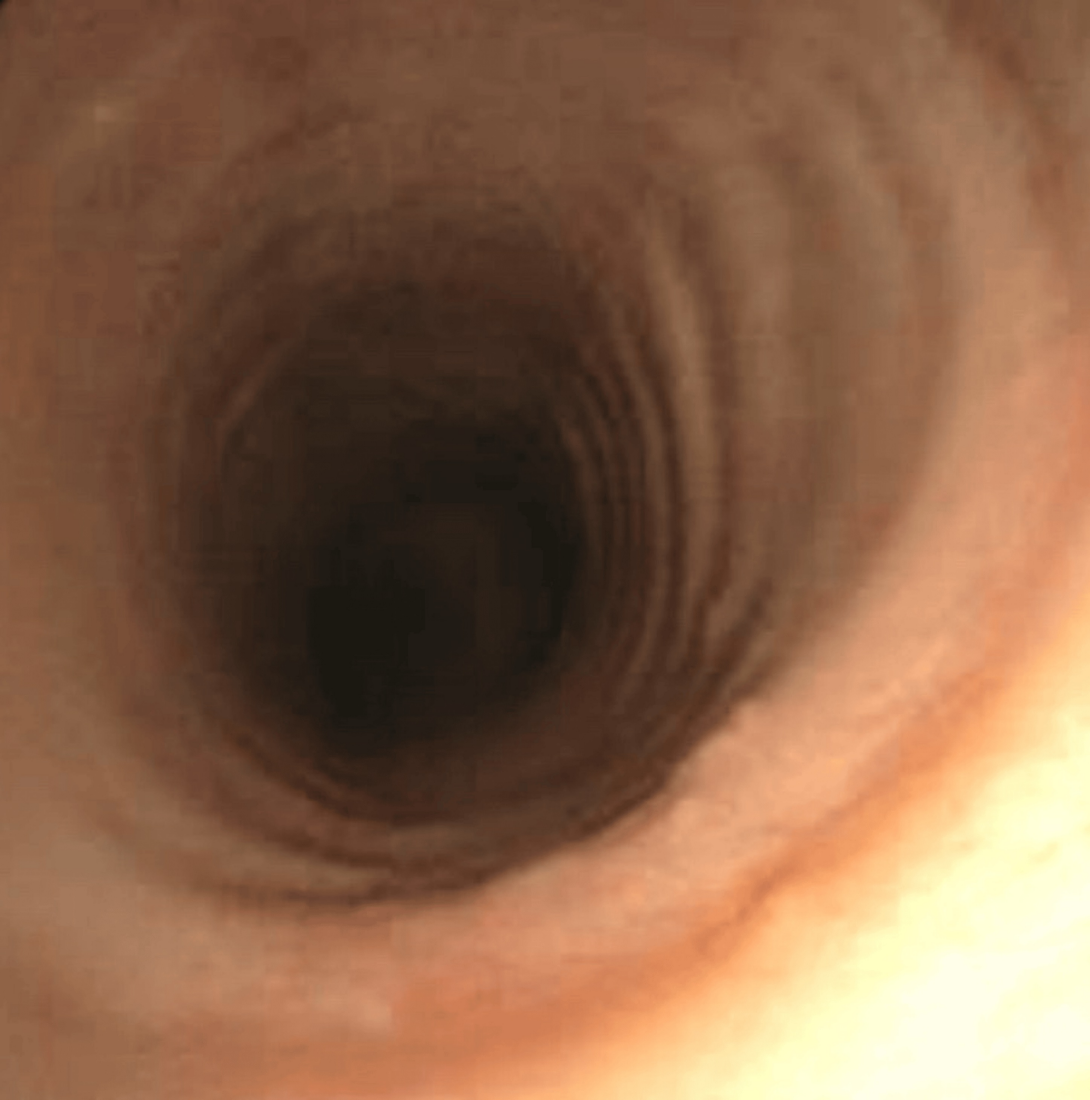
Bronchofibroscopy showing no lesions

A high-resolution chest CT scan revealed bilateral ground-glass opacities (Figure 2). Since the patient was a person living with HIV without any follow-up or treatment and the imaging studies were suggestive of PCP, empirical trimethoprim-sulfamethoxazole (250/1250 mg three times a day) and prednisolone (40 mg twice a day on the first five days, followed by 40 mg once a day in the next five days and 20 mg once a day on the following ten days) were started. BAL (Figure 3) fluid analysis was positive for *Pneumocystis jirovecii* (by PCR), *Cryptococcus neoformans* (by culture), and multidrug-resistant *Escherichia coli* (by culture). Additionally, liposomal amphotericin B (150 mg daily) plus flucytosine (1250 mg four times a day) and meropenem (1000 mg three times a day, directed to *Escherichia coli*) were added. Blood cultures collected were also positive for *Cryptococcus neoformans*.

**Figure 4:**
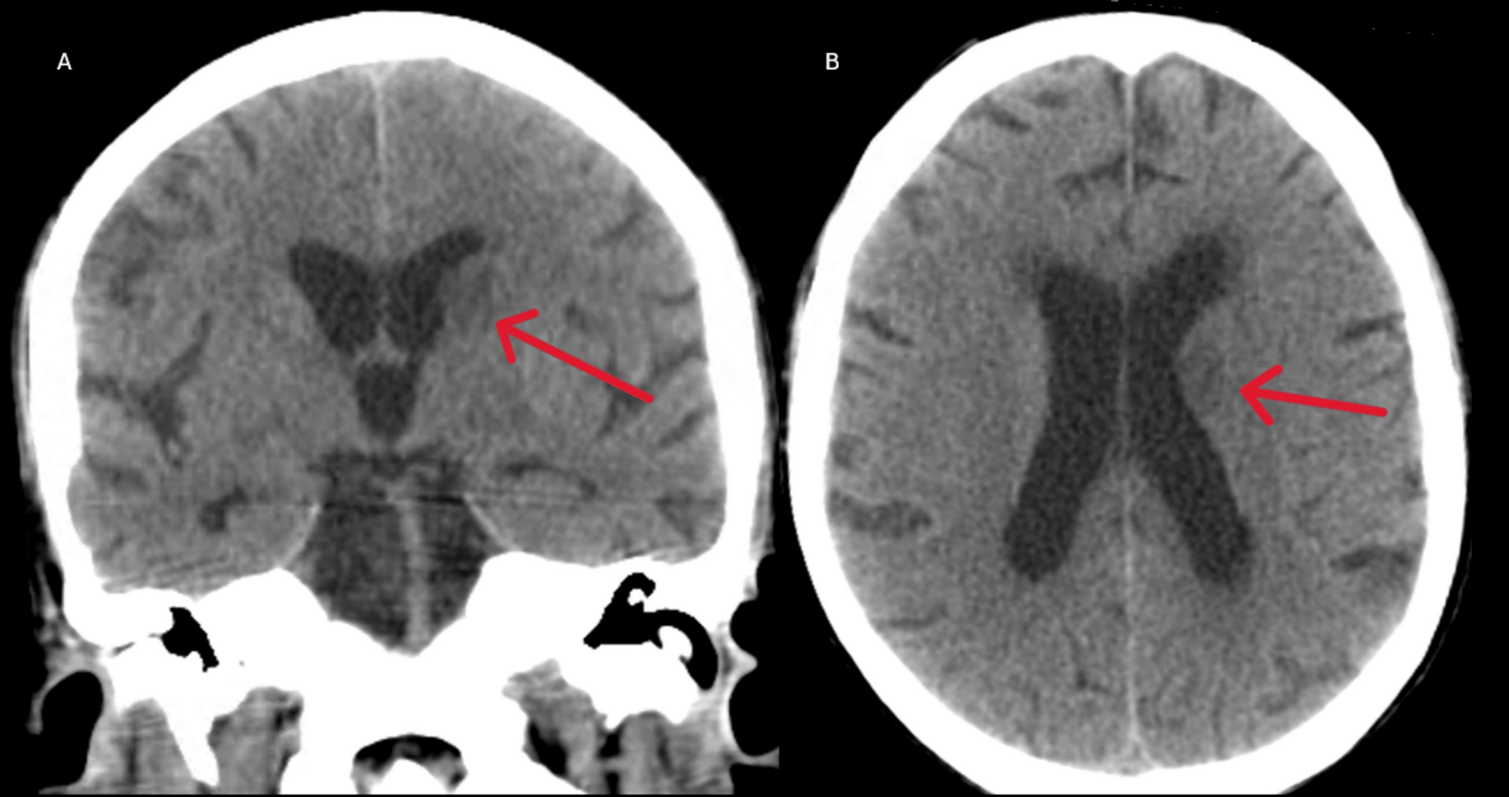
Coronal (A) and axial (B) view of the cerebral CT scan showing the hypodense area on the left gangliocapsular region (red arrows) CT: computed tomography

**Figure 5:**
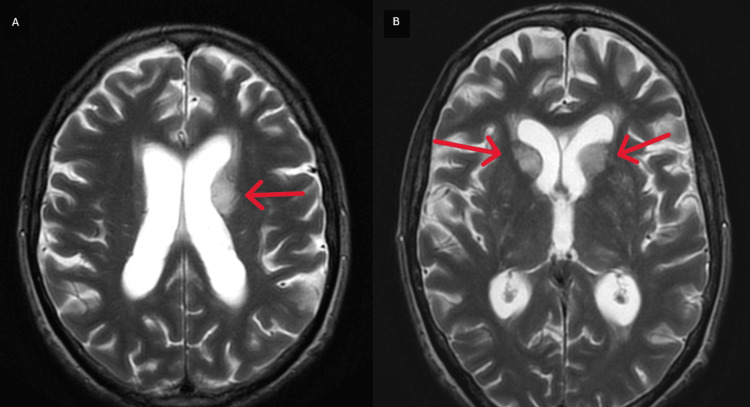
Axial view of the cerebral MRI T2 FSE showing the tumefactive lesions on the caudate nucleus bilaterally (red arrows) MRI: magnetic resonance imaging, FSE: fast spin-echo

Three days after admission, the patient started with headaches, nausea, vomiting, and lethargy, and a head CT scan was performed, showing hypodense areas in the left gangliocapsular region with mass effect (Figure 4A-4B). Cerebral MRI confirmed the presence of tumefactive lesions in the head of the right caudate nucleus and in the head and body of the left caudate nucleus, with hypersignal in T2. These lesions were compressing the frontal horns of the lateral ventricles (Figure 5A-5B) and were suggestive of cryptococcomas.

A lumbar puncture was performed (with an opening pressure of 21 cm H2O), yielding positive results for *Cryptococcus neoformans*, both antigen (titer >1:1024) and culture. Second and third lumbar punctures (opening pressure <20 cm H2O) were done on days 7 and 14 of treatment. Its results can be observed in Table 2.

**Table 2:** Lumbar puncture results

Parameter	First lumbar puncture	Second lumbar puncture	Third lumbar puncture	Normal range
Cells	1/uL	2/uL	35/uL	-
Proteins	45 mg/dL	61 mg/dL	150 mg/dL	15-45 mg/dL
Glucose	71 mg/dL	58 mg/dL	33 mg/dL	40-70 mg/dL

Due to the presence of *Cryptococcus neoformans* meningitis, the initiation of ART was deferred due to the risk of IRIS. Liposomal amphotericin B and flucytosine were administered for two weeks until the cultures were negative, followed by consolidation therapy with fluconazole (800 mg once a day). Regarding PCP, trimethoprim-sulfamethoxazole was maintained for 21 days, followed by secondary prophylaxis (160/800 mg thrice a week).

**Figure 6:**
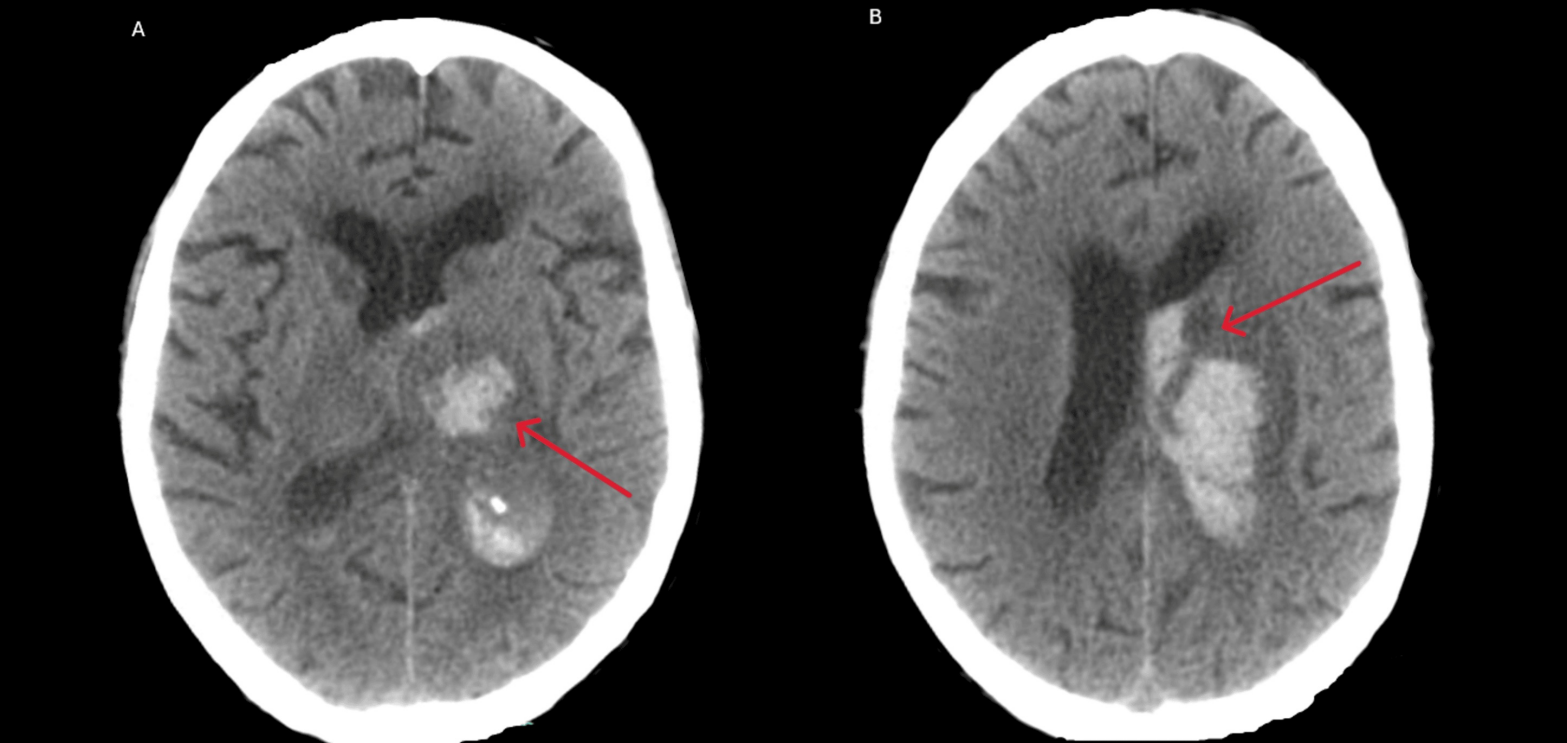
Axial view of the cerebral CT scan showing (A) hyperdensity in the corpus striatum, internal capsule, thalamus, and intraventricular area compatible with hemorrhage and (B) hemorrhagic lesion with signs of hydrocephalus CT: computed tomography

In the fourth week of hospitalization, the patient experienced a decreased level of consciousness, with a Glasgow coma scale of 10 (ocular response of three points, verbal response of two points, and motor response of five points), accompanied by vomiting, dizziness, headaches, right hemiparesis, and mixed aphasia. Emergent cerebral CT showed left gangliocapsular hyperdensity, compatible with hemorrhagic transformation following ischemic stroke, with ipsilateral intraventricular bleeding and hydrocephalus (Figure 6A-6B). He was subsequently transferred to the neurosurgery unit, where his condition deteriorated, not having surgical conditions and ultimately culminating in the patient's demise.

## Discussion

AIDS, or advanced HIV disease, is defined by a CD4 cell count <200/microL or the presence of any AIDS-defining condition regardless of CD4 cell count [1]. Several diseases are included as AIDS-defining conditions [1]. According to the literature, it takes about 12-18 months to develop an AIDS-defining condition [3]. However, our patient developed PCP and cryptococcosis 36 months after the initial HIV diagnosis.

*Pneumocystis jirovecii* is a leading cause of opportunistic infection in HIV-infected patients [6]. Our patient had a CD4 cell count of 1 cell/microL and a high HIV viral load, two of the major risk factors for developing PCP. As described in the literature [8], our patient displayed typical manifestations of PCP, including severe hypoxemia. High-resolution CT revealed bilateral ground-glass opacities, a main finding in these patients [11]. Due to the high suspicion of PCP, thanks to clinical and radiological features, empiric therapy with trimethoprim-sulfamethoxazole and corticosteroids was promptly started, as recommended [12]. BAL fluid PCR analysis is necessary to confirm the diagnosis [9], and in our patient’s case, it verified the presence of *Pneumocystis jirovecii*. Once confirmed, and according to the CDC guidelines [7], treatment was extended to 21 days.

In 15% of patients with PCP, a co-occurring infection is present [7]. BAL fluid culture in our patient revealed the presence of *Cryptococcus neoformans,* typically observed in patients with CD4 cell counts <100 cells/microL [7]. Cryptococcal infection often begins in the lungs, presenting with cough, dyspnea, and interstitial infiltrates mimicking PCP before disseminating to the CNS [14], presenting as meningitis or meningoencephalitis [7]. During hospitalization, our patient developed symptoms compatible with meningoencephalitis. Cerebral CT and MRI showed tumefactive lesions in the head of the right caudate nucleus and in the head and body of the left caudate nucleus, suggestive of cryptococcomas. In neurological cryptococcosis, CSF is characterized by a low white blood cell count with a lymphocytic predominance, mildly elevated protein levels, and low-to-normal glucose concentrations, as our patient presented. CSF culture and cryptococcal antigen culture confirmed the diagnosis of neurological cryptococcosis, as suggested by the literature [7]. Following CDC guidelines [7], our patient started induction therapy with liposomal amphotericin B plus flucytosine for two weeks until the CSF cultures were negative, followed by consolidation with fluconazole.

For *Pneumocystis jirovecii* infection, ART initiation should be deferred for two weeks, and for *Cryptococcus neoformans,* it should be deferred for four to six weeks due to the increased risk of IRIS [7]. Our patient did not initiate ART due to the development of a left gangliocapsular ischemic stroke with hemorrhagic transformation and hydrocephalus, necessitating transfer to the neurosurgery unit. Literature reports lacunar strokes in the basal ganglia as one of the main neurological complications of cryptococcal infection [16].

Survival rates for PCP and cryptococcosis improved with ART and targeted therapies [13-19]. For patients with advanced HIV infection, the median survival time is 12-18 months [5]. The patient ended up passing away; however, approximately 36 months went by between the initial HIV diagnosis and his death.

## Conclusions

Despite ART, AIDS-related opportunistic infections remain a reality. Opportunistic infections often can be present simultaneously. Cryptococcosis and PCP continue to be significant causes of mortality, especially in advanced HIV infection. Early diagnosis, targeted antifungal and antipneumocystis therapy, and ART initiation are fundamental to improving outcomes.
